# miRNA-221 and miRNA-222 synergistically function to promote vascular calcification

**DOI:** 10.1002/cbf.3005

**Published:** 2013-10-08

**Authors:** N C W Mackenzie, K A Staines, D Zhu, P Genever, V E MacRae

**Affiliations:** 1 Roslin Institute and Royal (Dick) School of Veterinary Studies, University of EdinburghEdinburgh, UK; 2 The Royal Veterinary CollegeLondon, UK; 3 Department of Biology, The University of YorkYork, UK

**Keywords:** calcification, Enpp1, microarray, miRNA, vascular smooth muscle cell

## Abstract

Vascular calcification shares many similarities with skeletal mineralisation and involves the phenotypic trans-differentiation of vascular smooth muscle cells (VSMCs) to osteoblastic cells within a calcified environment. Various microRNAs (miRs) are known to regulate cell differentiation; however, their role in mediating VSMC calcification is not fully understood. miR-microarray analysis revealed the significant down-regulation of a range of miRs following nine days in culture, including miR-199b, miR-29a, miR-221, miR-222 and miR-31 (*p* < 0.05). Subsequent studies investigated the specific role of the miR-221/222 family in VSMC calcification. Real-time quantitative polymerase chain reaction data confirmed the down-regulation of miR-221 (32.4%; *p* < 0.01) and miR-222 (15.7%; *p* < 0.05). VSMCs were transfected with mimics of miR-221 and miR-222, individually and in combination. Increased calcium deposition was observed in the combined treatment (two-fold; *p* < 0.05) but not in individual treatments. *Runx2* and *Msx2* expression was increased during calcification, but no difference in expression was observed following transfection with miR mimics. Interestingly, miR-221 and miR-222 mimics induced significant changes in ectonucleotide phosphodiesterase 1 (Enpp1) and *Pit-1* expression, suggesting that these miRs may modulate VSMC calcification through cellular inorganic phosphate and pyrophosphate levels. © 2013 The Authors. *Cell Biochemistry and Function* published by John Wiley & Sons, Ltd.

## INTRODUCTION

The pathological deposition of calcium phosphate mineral, most often hydroxyapatite, in arteries and cardiac valves has severe clinical consequences and is considered as an accurate predictor of future adverse cardiovascular events. Extensive research has shown that this vascular calcification is a tightly regulated process that shares many similarities with physiological bone mineralisation. Certainly, the trans-differentiation of vascular smooth muscle cells (VSMCs) into an osteo/chondrogenic phenotype has been defined, and along with subsequent changes in gene expression, this is thought to be the main mediator of vascular calcification. However, the precise mechanisms underlying the initiation and progression of pathological vascular calcification have yet to be fully determined.

MicroRNAs (miR) are an important class of endogenous, single stranded, non-coding RNAs, which are involved in the regulation of gene expression and translation, and as such, miRs are important in regulating cellular differentiation. This novel class of gene regulators are still however poorly understood; although more than 400 human miRs have been cloned, the biological functions of only a small portion have been characterized.^1^,^2^ miRs are important in the development, proliferation and function of VSMCs, and recent studies have defined a direct involvement of miRs in pathological calcification.^3^–^7^

In this study, we sought to establish which miRs are differentially regulated during the trans-differentiation and subsequent calcification of murine VSMCs through microarray analysis. We confirm that similar changes in miR expression identified in the microarray data can be detected by real-time quantitative polymerase chain reaction (RT-qPCR). We identify miR-221 and miR-222 as potentially important regulators of calcification, and as such, we transfected VSMCs with mimics of miR-221 and miR-222 revealing an increase in calcium deposition with concomitant changes in known regulators of mineralisation. Finally and somewhat surprisingly, we show that the effect of miR-221/222 was independent of the transcription factors *Runx2* and *Msx2*.

## MATERIALS AND METHODS

### Primary murine vascular smooth muscle cells isolation and maintenance

Primary VSMCs were isolated from 5-week old wild-type male C57BL/6 mice as previously described.^8^ Briefly, after removal of adventitia, the aorta was cut open to expose the endothelial layer. Tissues from 16 animals were pooled for digestion with 1 mg/ml trypsin for 10 min in order to remove any remaining adventitia and endothelium. Following a further overnight incubation at 37 °C in a humidified atmosphere of 95% air/5% CO_2_ in growth medium (*α*-MEM (Invitrogen, Paisley, UK) supplemented with 10% fetal bovine serum (Invitrogen) and 1% gentamicin (Invitrogen)), tissues were then digested with 425 U/ml collagenase type II (Worthington Biochemical Corporation, Lakewood, USA) for 5 h. Isolated VSMCs were expanded in growth medium for two passages in T25 tissue culture flasks (Greiner Bio-one, GmbH, Frickenhausen, Baden-Wurttemberg, Germany) coated with 0.25 µg/cm^2^ murine laminin (Sigma, Poole, UK) to promote maintenance of the contractile differentiation state.^9^

### Calcification of vascular smooth muscle cells for global miR expression analysis

Primary VSMCs were seeded in growth medium at a density of 1.5 × 10^4^/cm^2^ in multi-well plates (Corning Costar, MA, USA). At confluency (day 0), VSMCs were cultured in growth medium supplemented with 2.5 mM *β*-glycerophosphate (Sigma) and 50 µg/ml ascorbic acid (Sigma) to induce VSMC trans-differentiation and calcification as previously shown.^10^,^11^ RNA was extracted at day 9 of culture using the mirVana kit (Invitrogen) according to manufacturer's instructions.

### Affymetrix miR labelling, array hybridization and data processing

RNA quality was assessed by using the Agilent model 2100 bioanalyzer (Agilent Technologies, Palo Alto, CA). Only samples with intact, distinct ribosomal peaks were chosen for further analysis. Two micrograms of total RNA was processed for the microarray by using the Affymetrix flashtag RNA labelling kit (Affymetrix, Santa Clara, CA, USA) according to the manufacturer's recommended protocols by adding 2 µg total RNA to the tailing reaction (2.5 mM MnCl_2_, ATP and poly(A) polymerase) with incubation for 15 mins at 37 °C. This was followed by ligation of the biotinylated signal molecule to the target RNA sample (1× flash tag ligation mix biotin, T4 DNA ligase).

Each sample was hybridized to a GeneChip® miR array (Affymetrix) at 48 °C for 16 h. The arrays were washed, stained and scanned using the Affymetrix model 450 fluidics station and Affymetrix model 3000 scanner using the manufacturer's recommended protocols.

The image data were analysed with the miR quality control tool software for quality control software. Further analysis was performed using the GeneSpring GX10 software (Agilent Technologies, USA). The expression values were summarized and normalized respectively with robust multi-array average and variance stabilization method using robust multi-array average and variance stabilization method packages from Bioconductor 2.5.

### Transfection of microRNAs with microRNA mimics and culture under high phosphate conditions

Primary VSMCs were seeded in growth medium at a density of 1.5 × 10^4^/cm^2^ in multi-well plates (Corning Costar). Immediately, following seeding cells were transfected with 100 nM miScript miR mimics, in combination or individually, (miR-221: 5'AGCUACAUUGUCUGCUGGGUUUC3'; miR-222: 5'AGCUACAUCUGGCUACUGGGU3' and undisclosed Allstars negative control – Qiagen, West Sussex, UK) that had been complexed in 5 µl HiPerfect transfection reagent (Qiagen) and 45 µl Opti-Mem. medium (Invitrogen) for 20 mins. After 24 h, cells were cultured in control or high inorganic phosphate (P_i_) medium (α-minimal essential medium supplemented with 10% fetal calf serum, 1% gentamycin and 2 mM Na_2_HPO_4_/NaH_2_PO_4_ pH7.4), which we have previously shown to induce calcification after 7 days in culture.^11^ At day 3 in high P_i_ medium, the medium was changed and a repeat transfection mix was added to the fresh medium.

### Analysis of gene expression using quantitative real-time quantitative polymerase chain reaction

RNA was extracted from transfected cells using the RNeasy total RNA kit (Qiagen) or miRNeasy mini kit (Qiagen), according to the manufacturer's instructions at days 0, 3 and 7 of culture. For each sample, total RNA content was assessed by absorbance at 260 nm and purity by A260/A280 ratios. RNA was reverse transcribed using superscript II (Invitrogen) for total RNA and miRScript reverse transcriptase for miR preparations. Quantitative PCR (RT-qPCR) was performed using FastStart Sybr Green (Roche, East Sussex, UK) for total RNA and miScript Sybr Green PCR kit (Qiagen) on the Stratagene Mx3000P real-time RT-qPCR system (Stratagene, CA, USA).^12^,^13^ Primers for Runx2 (forward, 5ACCATAACAGTCTTCACAAATCCT3; reverse, 5CAGGCGATCAGAGAACAAACTA3), *Pit-1* (forward, 5'CACTCATGTCCATCTCAGACT3'; reverse, 5'CGTGCCAAAGAAGGTGAAC3') and ectonucleotide phosphodiesterase (*Enpp1*) *(*forward: 5'GCTAATCATCAGGAGGTCAAG3'; reverse, 5'CTGGTAGAATCCCGTCAATC3') were purchased from Primer Design (Southampton, UK). Sequences of Primers used to detect expression of *Gapdh* (Primer Design), Msx2 *(*Qiagen Ltd) and all miRs (Qiagen) were not disclosed by the manufacturer.

### Detection of calcification

The matrix was decalcified in 0.6 N HCL for 24 h, and free calcium determined colorimetrically using a commercially available kit (Randox Laboratories Ltd, County Antrim, UK) and corrected for total protein content measured using the Bio-Rad protein assay reagent (Bio-Rad Laboratories, Hertfordshire, UK). Gamma globulin was used as standard.^14^

### Statistical analysis

Data are presented as the means ± SEM. Statistical analysis was determined by the general linear model analysis incorporating pairwise comparisons and the student *t*-test using Minitab 15 (Minitab Inc, Coventry, UK). *p* < 0.05 was considered to be significant.

## RESULTS

### MicroRNAs regulate cellular changes during the trans-differentiation of vascular smooth muscle cells

**Figure 1:**
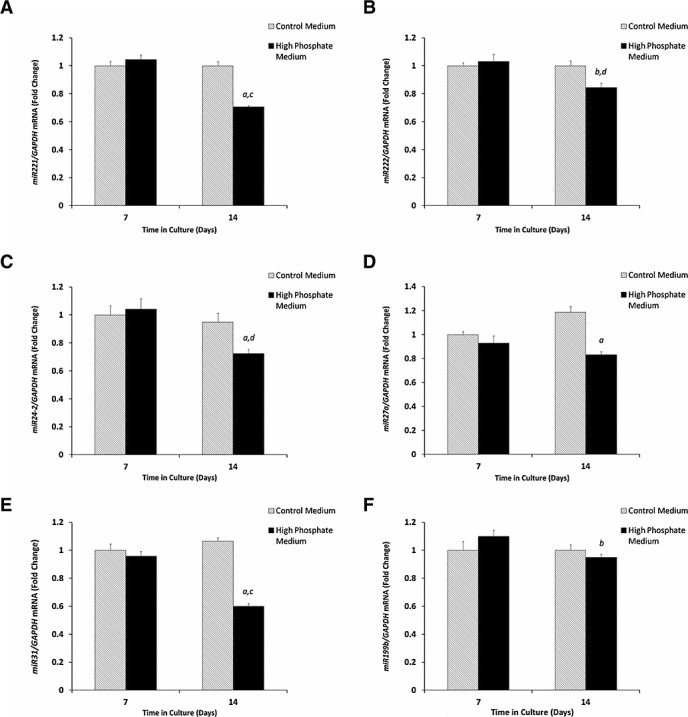
Down-regulation of microRNA expression during the *in vitro* calcification of murine aortic VSMCs cultured for 14 days with 3 mM P_i_ (high phosphate medium) in comparison with control medium. Fold change in the mRNA expression of (A) miR-221, (B) miR-222, (C) miR-24-2, (D) miR-27a, (E) miR-31 and (F) miR-199b. Results are presented as mean ± SEM, a) *p* < 0.01** versus day 7, b) *p* < 0.05* versus day 7, c) *p* < 0.01** versus control medium and d) *p* < 0.05* versus control medium

To determine the role of miRs in vascular calcification, we conducted miR microarray analysis of VSMCs cultured under calcifying conditions. This identified an extensive range of miRs differentially expressed during the trans-differentation of murine VSMCs in culture (>100), the most significant of which are detailed in Table 1. To confirm our microarray data, a selection of miRs was chosen for RT-qPCR validation. In agreement with the results of the microarray, these data indicated significant down-regulation of miR-221 (32.4%; *p* < 0.01), miR-222 (15.7%; *p* < 0.05), miR-24-2 (23.7%; *p* < 0.01), miR-27a (30%; *p* < 0.01), miR-31 (43.7%; *p* < 0.01) and miR-199b (13.6%; *p* < 0.05) expression in VSMC cells cultured for 14 days compared with 7 days in high P_i_ medium in culture (Figure 1A–F). Given that medial vascular calcification in humans is associated with high circulating phosphate levels, VSMCs were treated in a medium containing high P_i_, which we have previously shown to induce calcification *in vitro*.^11^ All miRs examined were also significantly down-regulated when cultured in high P_i_ medium in comparison with VSMCs cultured in control medium, apart from miR-199b (*p* < 0.05, Figure 1A–E), although the magnitude of change was considerably lower than the microarray study.

**Table 1:** MicroRNAs differentially expressed during the *in vitro* calcification of murine aortic VSMCs as analysed by microarray analysis

Down-regulated	
miRNA	Fold change
miR-379	222.5
**miR-199b**	269.8
let-7i	238
miR-16	192.5
miR-27b	195.1
miR-29a	168.2
**miR-221**	108.6
miR-199a	102.4
miR-674	139.2
miR-151	81.2
miR-100	95.3
**miR-222**	80.9
miR-22	90.3
miR-652	83.2
miR-130a	77.8
miR-382	69.7
miR-361	69.4
**miR-27a**	48.2
**miR-24-2**	43.8
**miR-31**	40.6
**Up-regulated**	
miR-706	19.3
miR-714	17.6
miR-1192	15.6
miR-487-b	8
miR-376b	9.7
miR-702	7.9
miR-703	6.8
miR-483	4.8
miR-208b	3.5
miR-759	3.1

Table shows those microRNAs that are significantly down-regulated or up-regulated after 9 days in calcifying culture medium when compared with day 0. Highlighted in bold are those selected for further examination.

miR, microRNAs.

### miR-221 and miR-222 synergistically act to promote vascular smooth muscle cells calcification

**Figure 2:**
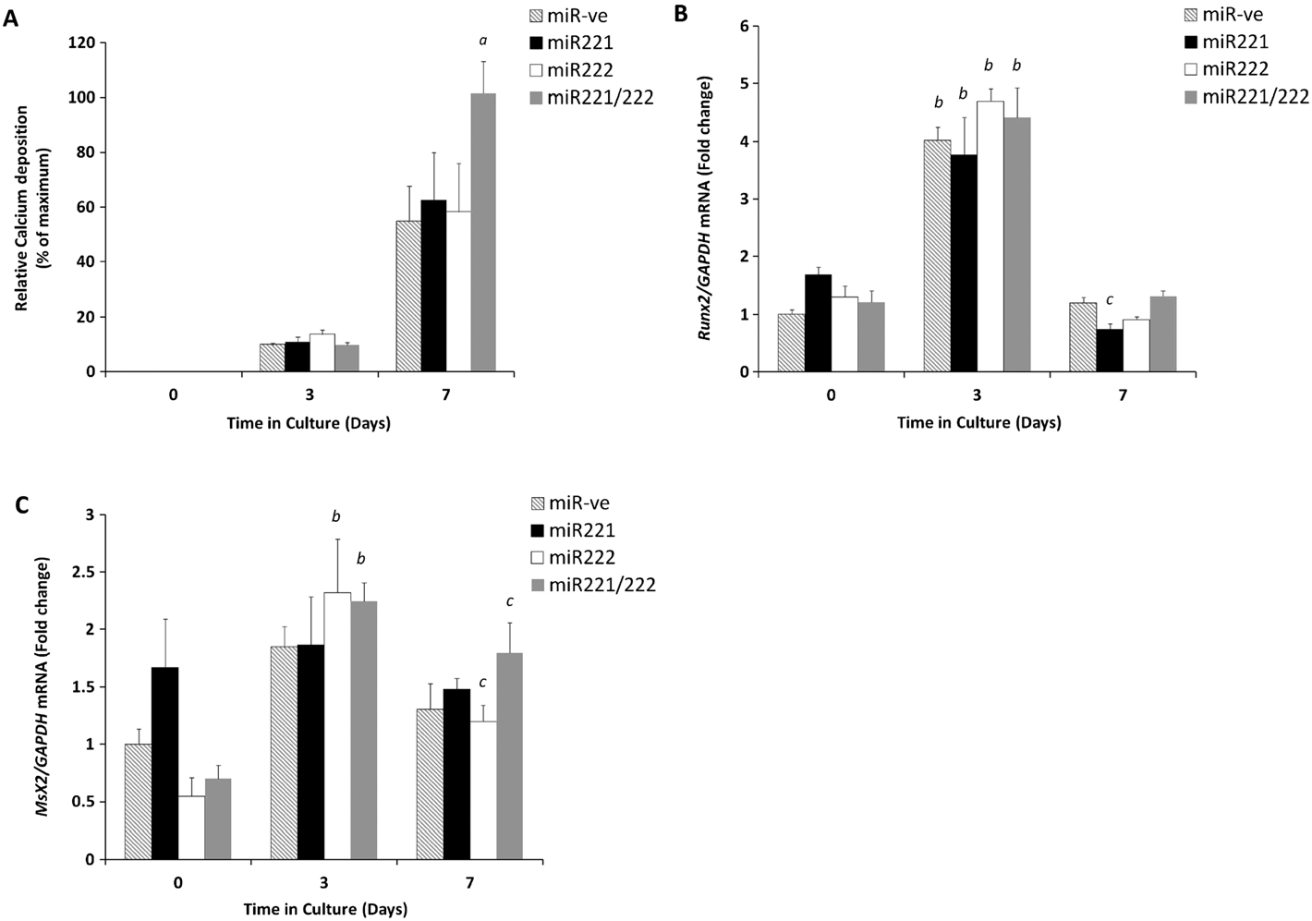
The effect of miR-221 and miR-222 transfections on the *in vitro* calcification of murine aortic VSMCs cultured for up to 7 days in high phosphate medium (3 mM P_i_). (A) Calcium content was determined by quantification of HCl leaching (microgram/milligramme protein) at days 3 and 7 of culture in comparison with day 0. Fold change in the mRNA expression of (B) *Runx2* (C) *Msx2*. Results are expressed as mean ± SEM, a) *p* < 0.05* versus miR-ve, b) *p* < 0.001*** versus day 0 and c) *p* < 0.05* versus day 0

Our microarray and RT-qPCR data confirmed that miR-221 and miR-222 are down-regulated during the calcification of murine VSMCs (Figure 1A and B). Because of the known roles of miR-221 and miR-222 in the cell cycle,^15^,^16^ we next sought to examine their functional role in VSMC calcification *in vitro*. We transfected VSMCs with mimics of miR-221 (50 nM) and miR-222 (50 nM), individually and in combination, alongside a miR-ve control transfection. All cell cultures showed time-dependent increases in calcium deposition after 7 days of treatment in comparison with days 0 and 3 of culture as expected (Figure 2A). Calcium deposition in VSMCs co-transfected with the combination of both miR-221 and miR-222 was significantly increased in comparison with those transfected with miR-ve (two-fold; *p* < 0.05, Figure 2A). Interestingly, cells transfected with individual miR-221 and miR-222 mimics did not show any significant differences when compared with the miR-ve treated cells (Figure 2A). These data suggest that the synergistic actions of miR-221 and miR-222 alter the trans-differentiation of VSMCs and increase the rate of calcification *in vitro*, which is not seen upon individual action.

### miR-221/222-induced VSMC calcification is independent of Runx2 and Msx2

The transcription factors Runx2 and Msx2 are pivotal in bone mineralisation; we and others have previously shown that Runx2 is critical during the trans-differentiation of VSMCs under high phosphate conditions.^11^,^17^,^18^ Therefore to examine whether miR-221 and miR-222 act through these transcription factors, VSMCs were treated with miR-221 and miR-222, in combination and individually, and were examined for Runx2 mRNA expression by RT-qPCR. Here, we found a significant increase in Runx2 mRNA expression in all VSMC cells following 3 days in high phosphate medium (in comparison with day 0, *p* < 0.001, Figure 2B). However, no significant differences were observed when the different combinations of miR treatments were considered (Figure 2B). Similarly, the osteogenic transcription factor Msx2, also showed increased mRNA expression at day 3 of VSMC culture. However, no significant differences were observed between cells treated with miR-221/222 in combination and cells treated with miR-ve (Figure 2C). These data suggest that the synergistic function of miR-221 and miR-222 in promoting vascular calcification is independent of Runx2 and Msx2.

### Altered expression of phosphate regulators by miR221/222

**Figure 3:**
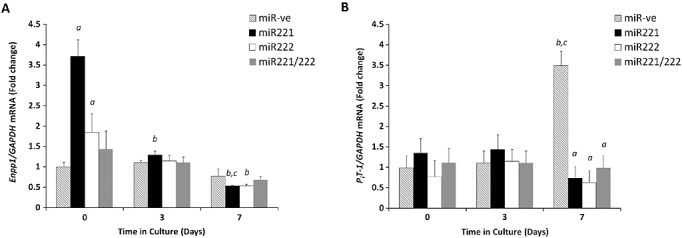
Changes in mRNA expression of known markers of VSMC calcification upon transfection with miR-221 and miR-222. Fold change in the mRNA expression of (A) *Enpp1* (B) *Pit-1*. Results are expressed as mean ± SEM a) *p* < 0.001*** versus miR-ve, b) *p* < 0.001*** versus day 0, c) *p* < 0.001*** versus day 3

Further studies examined the expression profile of Enpp1, which regulates vascular calcification through the generation of the mineralization inhibitor pyrophosphate (PP_i_).^19^,^20^ Twenty-four hours following transfection, prior to treatment with high phosphate medium (day 0), a significant increase in Enpp1 mRNA expression in VSMCs transfected with either miR-221 or miR-222 was observed when compared with miR-ve transfected cultures (Figure 3A, *p* < 0.001). In contrast, VSMCs transfected with both miR-221/222 showed no significant differences in comparison with miR-ve transfected cultures (Figure 3A). These findings may offer some explanation to the differences in VSMC calcification observed between individual and combined transfection. Furthermore, in control cells transfected with miR-ve, there was a significant increase in expression of type III sodium-dependent P_i_ cotransporter-1 (*Pit-1*) on day 7 of culture in high phosphate medium, as previously reported in studies from this group.^10^ These increases were not observed in VSMCs transfected with miR-221 and/or miR-222 (Figure 3B, *p* < 0.001). Taken together, these data indicate that the miR-221 and miR-222 may lead to changes in the cellular balance of P_i_ and PP_i_ through altered Enpp1 and *Pit-1* expressions.

## DISCUSSION

Despite recent advances in our knowledge, the full mechanisms underpinning vascular calcification have yet to be fully elucidated. This study has clearly demonstrated differential expression of novel miRNAs in murine primary vascular smooth muscle cell cultures, cultured under calcifying conditions. Of note, a wide range of miRs associated with cell proliferation, differentiation and oncogenesis has been shown to be down-regulated through microarray analysis. We selected a small number of miRNAs and use RT-qPCR to confirm their down-regulation in VSMCs under high phosphate culture conditions, designed to mimic high levels of circulating phosphate associated with medial calcification.^21^ These data confirm the significant down-regulation of miR-221, miR-222, miR-24-2, miR-27a and miR-31 during *in vitro* vascular calcification. The differences observed between the magnitude of down-regulation in the microarray and RT-qPCR studies are likely due to the different methods of analysis and normalization tools used.

A number of miRs associated with VSMC calcification in the present study have previously been implicated in cancer development.^22^–^25^ In particular, miR-24-2 and mir-27a have been shown to constitute a cluster, along with miR-23a, which has known roles in the promotion of cell differentiation in several cancers.^26^,^27^ More recently, this cluster has also been implicated in the repression of genes involved in the inhibition of bone formation. Both a feed-forward mechanism by which Runx2 acts to suppress this cluster and in turn prevent their inhibitory effects on bone formation and a feedback mechanism by which miR-23a directly inhibits Runx2 expression have recently been reported.^28^ This therefore suggests that this miR cluster acts to inhibit the differentiation of the osteoblast into an osteocyte. The similarities between osteoblast matrix mineralisation and VSMC calcification, as well as the known trans-differentiation of the VSMC into an osteoblast/osteocyte phenotype,^10^ are therefore consistent with the down-regulation of this cluster during the vascular calcification process.

We have also confirmed the down-regulation of miR-31 during *in vitro* VSMC calcification, which too has been implicated in the inhibition of osteogenic differentiation. Microarray analysis of differentiating mesenchymal stromal cells has recently highlighted a potent functional role for miR-31, involving the inhibition of tissue non-specific alkaline phosphatase (TNAP) activity through a mechanism dependent on Runx2 and *Bmp2*.^29^ Comparable findings have also been recently observed in bone marrow stromal cells and in the trans-differentiation of the VSMC phenotype through the cellular repressor of E1A-stimulated genes.^30^–^32^ Additionally, miR-31 has been shown to be increased in the serum of patients with coronary artery disease, which present with severe vascular calcification.^30^ This report of miR-31 as a potential biomarker in a disease involving VSMC phenotype modulation is likely to have major clinical benefit. These clinical data highlight the potential for further miRs to be investigated as biomarkers of vascular calcification, including those identified in the present study.

This study presents the first evidence to suggest that miR-221 and miR-222 contribute to the pathological process of vascular calcification. We have demonstrated the down-regulation of these miRs during VSMC calcification, which is consistent with recent microarray analysis of differentiating mesenchymal stromal cells.^29^ We also reveal a novel functional role for the synergistic actions of miR-221 and miR-222 in VSMC calcification under high phosphate conditions. Through the transfection of calcifying VSMCs with mimics of these miRs, we show that there are no significant changes in Runx2 and Msx2 expressions between different miR treatments. These data indicate that the increases in calcification observed are independent of these potent transcription factors and are consistent with the trans-differentiation of the VSMCS to an osteoblast/osteocyte phenotype as has previously been described.^10^,^11^,^33^ It is interesting to note that whilst we see a down-regulation of miR-221 and miRNA-222 in calcifying cells, the increase in cellular miR levels synergistically act to increase levels of cellular calcification. We hypothesize that these miRs may be involved in the initiation of early stages of cellular trans-differentiation and that their expression is reduced as the cells progress towards an osteo/chondrogenic phenotype. This theory is supported by studies on human stem cells showing that miR222 was up-regulated during the initiation of differentiation to neuronal cells and subsequently reduced as the cells reached adulthood,^34^,^35^ as well as recent investigations demonstrating that the down-regulation of miR221 induces osteogenic differentiation.^36^ Further studies into the effects of these miRs on VSMCs cultured under low phosphate conditions may provide new insights into their role during the initiation of differentiation.

Furthermore, we have shown that miR-221 and miR-222 may regulate the cellular balance of P_i_ and PP_i_ through altered Enpp1 and *Pit-1* expression. This suggests a mechanism through which these miRs may regulate VSMC trans-differentiation and calcification. This is supported by recent studies, which have highlighted these molecules as key regulators of vascular calcification.^10^,^20^,^21^,^37^ Our data show that the initial transfection with individual miRNAs induces an increase in Enpp1 expression at day 0 (24 h after transfection), which is subsequently reduced following the widespread changes in gene expression seen following high P_i_ treatment.^11^,^37^ It is likely that these changes in Enpp1 expression at day 0 are due to the trans-differentiation of cells towards an osteogenic phenotype and associated changes in gene expression. Interestingly, the increase of Enpp1 expression is not observed in the combined miR-221/miR-222 treatments, which may give an explanation as to the higher level of calcification seen in these cells and suggests that the changes in gene expression may be regulated by upstream pathways. Indeed, we have found no apparent evidence of miR-221 or miR-222 directly targeting any of the known regulators of cellular P_i_/PP_i_ concentrations such as *Enpp1, Alpl, Ank, Pit-1 and Phospho1*.^20^,^37^ In fact, the previously documented promotion of vascular calcification by *Pit-1* suggests that the effects we are seeing here are not due to direct increases in *Pit-1* *per se*.^38^ It is conceivable that the changes in Enpp1 and *Pit-1* observed in this study are a result of upstream changes in cell cycle progression mediated by transfection of cells with miR-221 and/or miR222, with combined miRNAs acting on a wider range of pathways than the individual miRNAs. It has recently been shown that induction of the cAMP/protein kinase A pathway significantly alters the expression of Enpp1, ANK and TNAP during phosphate induced calcification.^39^ Further studies into the pathways leading to changes in NPP1 and *Pit-1* and subsequent effects on the cellular and extracellular levels of P_i_ and PP_i_, would be very revealing. It would also be of interest to expand these investigations to additional regulators of production, transport and degradation of P_i_/PP_i_.

miR-221 and miR-222 have been previously described as regulators of cell cycle progression via p21, p27kip1 and p57 kip2 in various human cancers.^15^,^16^,^40^,^41^ Furthermore, they have been implicated in tumour necrosis factor-related apoptosis-inducing ligand (TRAIL) resistance in lung cancer cell lines through interfering with p27kip1, phosphatase and tensin homology and tissue inhibitors of metalloproteinases expressions.^41^–^43^ Consistent with their role in cancer cells, miR-221 and miR-222 have been shown to have pro-proliferative, pro-migratory and anti-apoptotic effects that effect on cultured VSMCs.^44^ This increase in proliferation has been partially attributed to changes in p27(kip1) and p57(kip2); however, the full effects of these miRs in VSMCs have yet to be fully determined. miR-221 has also been shown to regulate chondrogenic differentiation by targeting *Mdm2*, further highlighting the potential importance of these miRs in the calcification process.^45^

In conclusion, we have established that miR-221 and miR-222 work concomitantly to alter the trans-differentiation of murine VSMCs and promote calcification *in vitro*. We identify a potential mechanism of action through the calcification regulators Enpp1 and *Pit-1*. These data will provide advances towards clinical benefit by offering a greater understanding of the molecular mechanisms underpinning vascular calcification, an acute predictor of future adverse cardiovascular events.
